# Chemistry General, Medical, and Pharmaceutical, Including the Chemistry of the U. S. Pharmacopœia

**Published:** 1871-06

**Authors:** 


### Book Notices.

Chemistry General, Medical, and Pharmaceutical, including The Chemistry of the U. S. Pharmacopœia. A Manual on the General Principles of the Science, and their Application to Medicine and Pharmacy. By John Attfield, Ph. D., F.C.S. From the second and enlarged English edition. Published by Henry C. Lea, Philadelphia. For sale by Cobb Bro., 81 and 83 Lake St., Chicago.

This is a small work of 500 pages, intended especially to meet the wants of medical and pharmaceutical students and practitioners. It differs from other text-books, first, in the exclusion of matter relating to compounds, which at present are of interest principally to the scientific chemist; secondly, in containing the chemistry of every substance recognized officially, or in general practice, as a remedial agent; and thirdly, in the paragraphs being so cast that the volume may be used as a guide in studying the science experimentally. A comprehensive index, containing five thousand references, renders the work a valuable one for consultation in the course of business or professional practice. The chemical notation of the work is in accordance with modern theories. The metric system of weights and measures is alone used in the sections on quantitative analysis.

